# The Regulatory Roles of Chemerin-Chemokine-Like Receptor 1 Axis in Placental Development and Vascular Remodeling During Early Pregnancy

**DOI:** 10.3389/fcell.2022.883636

**Published:** 2022-05-17

**Authors:** Qingqing Zhang, Zhonglin Xiao, Cheuk-Lun Lee, Yong-Gang Duan, Xiujun Fan, William S. B. Yeung, Philip C. N. Chiu, Jian V. Zhang

**Affiliations:** ^1^ Shenzhen Key Laboratory of Fertility Regulation, The University of Hong Kong-Shenzhen Hospital, Shenzhen, China; ^2^ Department of Obstetrics and Gynecology, The University of Hong Kong, Hong Kong, Hong Kong SAR, China; ^3^ Center for Energy Metabolism and Reproduction, Shenzhen Institute of Advanced Technology, Chinese Academy of Sciences, Shenzhen, China; ^4^ Shenzhen Institute of Advanced Technology, Chinese Academy of Sciences, Shenzhen, China; ^5^ Shenzhen Key Laboratory of Metabolic Health, Shenzhen, China

**Keywords:** chemerin, CMKLR1, placental development, trophoblast invasion, spiral artery remodeling

## Abstract

Chemerin is an adipokine that regulates metabolism in pregnancy. An elevation of serum chemerin level is associated with pregnancy complications. Consistently, we demonstrated that the chemerin expression was increased in placenta of preeclamptic patients at deliveries. The G protein-coupled receptor chemokine-like receptor 1 (CMKLR1) mediates the actions of chemerin. The functions of the chemerin-CMKLR1 axis in maintaining pregnancy are still unknown. In this study, we demonstrated that CMKLR1 was expressed in the decidual natural killer (dNK) cells and chorionic villi of human. Chemerin suppressed the proliferation of the dNK cells *in vitro*. Specific antagonist of CMKLR1, α-Neta abolished the suppressive effect of spent medium from chemerin-treated dNK cells culture on extravillous trophoblast invasion. Activation of the chemerin-CMKLR1 axis promoted fusion and differentiation of human cytotrophoblast to syncytiotrophoblast *in vitro*. We generated *Cmklr1* knockout mice and showed that the *Cmklr1* deficiency negatively affected pregnancy outcome in terms of number of implantation sites, litter size and fetal weight at birth. Histologically, the *Cmklr1* deficiency impaired formation of the syncytiotrophoblast layer II, induced enlargement of the maternal lacunae in the labyrinth, increased the diameter of the spiral arteries and increased trophoblast invasion in the decidua. The *Cmklr1* deficient placenta also displayed an increased number of dNK cells and serum IL-15 level. In summary, the chemerin-CMKLR1 axis regulated placental development and spiral artery remodeling in early pregnancy.

## Introduction

Successful pregnancy depends on proper formation of the placenta, which forms the site of maternal-fetal exchange for nutrients and wastes (King et al., 2000; Ahokas and McKinney, 2008). The placenta comprises of trophoblastic layers with distinct ontogeny and function (Marsh and Blelloch, 2020). In mice, these layers arise from the spongiotrophoblast, trophoblast giant cells, and syncytiotrophoblasts in the labyrinth (Rossant and Cross, 2001). The development of labyrinth is initiated by fusion of the chorion with the allantois at around E8.5 (Cross et al., 2003), followed by invagination of the allantoic capillaries into the chorionic trophoblast layer that triggers the differentiation of syncytiotrophoblasts (Woods et al., 2018). Then the extensive process of branching morphogenesis forms an intricate network of syncytiotrophoblast-lined maternal blood spaces and allantoic mesoderm-derived fetal components of the placental vascular network (Maltepe and Fisher, 2015).

Trophoblast invasion is crucial to placentation which is achieved by the orchestrated effort of various cell types such as dNK cells, macrophages, and extravillous trophoblasts (EVTs) (Lyall, 2005; Whitley and Cartwright, 2009). After implantation, the cytotrophoblasts differentiate into EVTs and form the migratory cell columns that invade the decidua and contribute to the spiral arteries remodeling (Hiden et al., 2007). Disruption of this process in humans impairs placental blood flow and is associated with pregnancy complications such as recurrent pregnancy loss, fetal growth restriction, and preeclampsia (PE) (Jim and Karumanchi, 2017; Knöfler et al., 2019). *In vivo* studies demonstrated that the EVTs directly interact with the dNK cells during EVT invasion (Tilburgs et al., 2015), suggesting that their functions are modulated in the interactions (Co et al., 2013; Pollheimer et al., 2018). In addition, multiple dNK cells-derived factors regulate the EVTs-related processes *in vivo* as well as *in vitro* (Lash et al., 2006; Zhang et al., 2013; Pollheimer et al., 2018). However, much of the molecular mechanisms on the actions of decidual immune cells on EVT invasion and spiral artery remodeling remain unknown.

Chemerin, also known as tazarotene-induced gene 2 (TIG2), is secreted by various cell types, including adipocytes, epithelial and endothelial cells (ECs), fibroblasts, and trophoblasts (Carlino et al., 2012; Helfer and Wu, 2018; Xie et al., 2020). Chemokine-like receptor 1 (CMKLR1, encoded by the *Cmklr1* gene), also known as ChemR23, is a G protein-coupled receptor and the natural receptor of chemerin (Mariani and Roncucci, 2015). CMKLR1 is widely expressed in the whole body including the placenta, ECs, and immune cells (Carlino et al., 2012; Zabel et al., 2014; Cetin et al., 2017; Yang et al., 2018). Chemerin-CMKLR1 axis participates in various biological responses such as inflammation (Xie et al., 2020), insulin resistance (Sell et al., 2009), chemotaxis, endocytosis, and carbohydrate/fat metabolic process (Yoshimura and Oppenheim, 2011; Helfer and Wu, 2018). The serum levels of chemerin are notably increased during pregnancy, suggesting that the chemerin-CMKLR1 signaling may play a regulatory role in the process (Yang et al., 2018). Besides, the concentration of chemerin in maternal serum is significantly higher in preeclamptic patients than in normal pregnant women (Spradley et al., 2015; Cetin et al., 2017). However, the *in vivo* biological functions of chemerin-CMKLR1 in the maintenance of early pregnancy and placental development have not been elucidated.

To understand the role of the chemerin-CMKLR1 signaling in pregnancy, we generated *Cmklr1* knockout mice and demonstrated its regulatory roles in embryonic and placental development. In this study, we also investigated the role of the chemerin-CMKLR1 axis in trophoblast invasion, dNK cells proliferation, syncytiotrophoblast fusion, and differentiation *in vitro*.

## Materials and Methods

### Immunohistochemistry of Chemerin for Human Placenta

The sections of term placentas of normal pregnancy and PE patients were subsequently de-waxed, rehydrated, the antigen was retrieved by using a 1× Universal HIER antigen retrieval reagent (ab208572, Abcam, MA 02453, United States) and incubated with 3% H_2_O_2_. Sections were then blocked with 10% goat serum (ab7481, Abcam) for 1 h at room temperature. The sections were incubated in chemerin (1: 100, ab72965, Abcam) overnight in the cold room. Chemerin staining was visualized using a DAB substrate kit for peroxidase from Vector Laboratories and counterstained and mounted as described above. Digital photographs at 4× and 10× were taken, and Image J software was used to quantify the H-score.

### Isolation of Primary Human dNK Cells

This study was approved by the Institutional Review Board (IRB) of The University of Hong Kong/Hospital Authority Hong Kong West Cluster (IRB No: UW 17-057). Human dNK cells were isolated as described (Male et al., 2012; Lee et al., 2018) from late first-trimester decidual tissues collected from patients who had undergone surgical termination of pregnancy due to psychosocial reasons with written consent. The tissues were minced and digested with collagenase (300 U/ml) and DNase I (50 μg/ml), and the cells were passed through 100 and 40 μm filters followed by Ficoll-Paque (17,144,002, GE Healthcare Illinois, United States) density gradient centrifugation. The cells were then cultured on a plastic plate overnight. The non-adherent cells were collected, and dNK cells were further enriched using CD56 microbeads (Miltenyi Biotec, Germany). The isolated dNK were cultured with RPMI medium supplemented with 10% FBS and 1% penicillin-streptomycin under standard culture conditions in a 37°C and 5% CO_2_ incubator.

### Determination of Proliferation and Cytokine Secretions of Human dNK Cells

The CyQUANT^®^ cell proliferation assay (CyQUANTTM NF Cell Proliferation assay kit, C35006, Invitrogen) was employed as a measurement of cell proliferation according to the manufacturer’s instructions. Primary dNK cells (5 × 10^3^ cells/well) were seeded in 96 well plate and cultured for 24 h with 0.5 µM chemerin (SRP6002, Sigma, St. Louis, UnitedStates), or 0.5 µM chemerin +1 µM 2-(alpha-Naphthoyl)ethyl trimethylammonium iodide (α-Neta, sc-221190A, Santa Cruz Biotechnology, United States) in RPMI1640 incomplete medium with 500 U/ml interleukin (IL)-2. The fluorescence intensity was measured using a fluorescence microplate reader (Infinite F Nano^+^) with an excitation wavelength at 485 nm and an emission wavelength at 530 nm. The concentrations of IL-8, IL-10 and TNF-α in the spent medium of dNK cells were determined using commercially available enzyme-linked immunosorbent assay (ELISA) kits (KHC3012, BMS215-2 and KHC0081, Abgent, San Diego, CA, United States). The samples were analyzed in quadruplicate.

### *In Vitro* Models to Study Human Trophoblast Invasion

JEG-3 cells (HTB-36, ATCC) were cultured in DMEM/Ham’s F-12 medium (Sigma, St. Louis, MO) containing 10% fetal bovine serum (FBS) and 1% penicillin-streptomycin, at 37°C, in an atmosphere of 5% CO_2_. The trophoblast invasion was measured by invasion assay (BD BioCoat™ Matrigel™ Invasion Assay, BD Biosciences). In brief, JEG3 cells (1 × 10^4^ cells/well) were placed in the upper invasion chamber, with the spent medium from dNK cells (1 × 10^5^ cells/well) treated with 0.5 µM chemerin, or 0.5 µM chemerin + 1 µM α-Neta treatment for 24 h were added into the upper chamber. FBS (10%) was added as a chemoattractive agent in the lower chamber. The invaded cells on the lower membrane of the chamber were stained with crystal violet, and images were acquired under light microscopy. The cells were then dissolved by 10% acetic acid, and the absorbance was measured at 595 nm.

### *In Vitro* Models to Study Human Trophoblast Fusion and Differentiation

The BeWo cell line has been widely used as an *in vitro* model for studying trophoblast intercellular fusion and differentiation (Heaton et al., 2008). BeWo cells (CCL-98, ATCC) were cultured in DMEM/Ham’s F-12 medium (Sigma, St. Louis, MO) containing 10% FBS and 1% penicillin-streptomycin, at 37°C, in an atmosphere of 5% CO_2_. BeWo cells (1 × 10^6^ cells/well) were plated in 6 well multi-dishes and maintained in culture until the cells were 70–80% conﬂuent. Differentiation was induced by the addition of 20 µM forskolin (F6886, St. Louis, MO, as positive control) with or without 0.5 µM chemerin, or 0.5 µM chemerin +1 µM α-Neta, in a serum-free medium for 48 h. All experiments involving the cell treatment were accompanied in parallel by vehicle controls. The quantification of BeWo cell fusion under different stimulatory conditions was performed by calculating the fusion index in 20 randomly selected microscopic fields of each condition. The fusion index = number of nuclei in syncytia/total number of nuclei x 100%. The cells were harvested to measure the expression of relevant differentiation markers by qPCR.

### Semi-Quantitative RT-PCR

Total RNA was extracted from placentas or BeWo cells after 48 h of treatment using Illustra RNAspin Mini RNA isolation kit (25-0500-71, GE Healthcare, United States). RT-PCR was performed following the protocol for Taqman Gold RT-PCR (Manufacturer). For cDNA amplifications, highly specific forward and reverse primers were used with initial heating at 95°C for 10 min, followed by 40 cycles of 95°C for 15 s and 60°C for 1 min. The TaqMan™ Gene Expression Assay primers were purchased from Life Technologies, including mouse Interleukin (IL)-6 (Mm00446191_m1); IL-8 (Mm04208136_m1); IL-10 (Mm01288386_m1); Tumor necrosis factor-alpha (TNF-α) (Mm00443258_m1); Matrix metalloproteinase-2 (MMP)2 (Mm01253624_m1); MMP9 (Mm00600157_g1); Human Glial Cells Missing-1 (GCM1) (Hs00961601_m1); syncytin-1 (Hs00835189_CE); Caudal-type homeobox gene 2 (CDX2) (Hs01078080_m1); Human leukocyte antigen G (HLA-G) (Hs00365950_g1); and CK7 (Hs00559840_m1).

### Mouse Model

C57BL/6 mice (8 weeks) were obtained from the Guangdong Medical Laboratory Center (Guangdong, China). *Cmklr*1^*−/−*^ mice were obtained from The Jackson Laboratory (Bar Harbor, ME United States). Estrous *Cmklr1*-deficient females were paired with *Cmklr*1^*−/−*^ males to obtain timed pregnancy, and copulation plug detection the next morning was designated as gestation day (GD) 1. The same procedure was performed between wildtype mice. Animals were housed in a controlled environment where constant temperature, humidity, and a 12-h light-dark cycle with free access to chow diet and water were given. All animal usage has complied with the procedures approved by the Committee on the Use of Live Animals for Teaching and Research, Shenzhen Institutes of Advanced Technology, Chinese Academy of Sciences (Permit Number: SIAT-IRB-120223-A0009).

### Measurement of Placental Layers

Undissected GD 12 mice enveloped within uterine tissue were collected. Tissue samples were fixed in fixative solution (Pyrocitric acid: formaldehyde: glacial acetic acid 15: 5: 1) and processed for embedding in paraffin. Paraffin-embedded tissues were sectioned into 5 µm for hematoxylin and eosin staining. Digital photographs of three central sections of each placenta were taken. Image J software was used to measure the relevant stained or unstained regions of each section. For each placental section, the area of the whole placenta, labyrinth, junctional, and decidua zone was measured.

### Lectin Histochemistry for Fetal Blood Vessels and Mice dNK

The sections of GD12 placenta from both wildtype and *Cmklr*1-deficient pregnant mice mounted on glass slides were subsequently de-waxed, rehydrated, and incubated with 3% H_2_O_2_. It was then washed three times with PBS, and the antigen was retrieved by using a 1× Universal HIER antigen retrieval reagent (ab208572, Abcam, MA 02453, United States). Sections were then blocked with 10% goat serum (ab7481, Abcam) for 1 h at room temperature. The sections were incubated in isolectin B4 (BSI-B4)(1: 100 in PBS, L-5391, Sigma-Aldrich, St Louis, United States) for fetal blood vessels and biotinylated-Dolichos biflorus (DBA) lectin (1: 1,000 in 1% BSA/PBS, L6533, Sigma Aldrich, Saint Louis, MO, United States) for dNK. The sections were incubated for 1 h and then washed three times in PBS. The lectin binding was detected by 30 min incubation with streptavidin-peroxidase (Extraditing-peroxidase; E2886, Sigma Aldrich, Saint Louis, MO, United States) and peroxidase-labeling kit (Vector Laboratories, city, country). The slides were then counterstained with hematoxylin, and then mounted followed by dehydration and clearing through a graded series of ethanol and xylene washes.

### Immunofluorescent Staining of MCT4 for Syncytiotrophoblast II Development and TPBPA for the Glycogen Trophoblast Cells, Giant Cells and Spongiotrophoblasts in the Junctional Zone

For immunostaining, antigen retrieval was performed in the deparaffinized sections as described above. The sections were permeabilized by eBioscience™ Permeabilization Buffer (00-8333-56, Thermo Fisher Scientific, Massachusetts, United States), followed by blocking in 10% goat serum (ab7481, Abcam) for 1 h. The sections were incubated with anti-monocarboxylate transporter 4 (MCT4, 1: 100, AB3314P, Merck Millipore) or anti-trophoblast specific protein alpha (TPBPA, 1: 100, ab104401, Abcam) antibodies overnight. After incubation, sections were washed three times in PBS. Primary antibodies were detected with appropriate fluorescence-conjugated secondary antibodies (1: 500 dilutions in PBS) for 1-h incubation. After incubation, sections were washed by PBS three times for 15 min. Nuclei were then counterstained with 4’,6-Diamidino-2-phenylindole dihydrochloride (DAPI, 1:500 dilution in PBS, D9542, MERCK, United States) for 2 min followed by washing with PBS five times. The sections were then mounted with a fluorescence mounting medium (S3023, DAKO, United States) and digital images were captured under a fluorescent microscope.

### Immunohistochemistry of Cytokeratin 8 for Trophoblast

The sections were incubated with, anti-CK8 antibody (ab53280, Abcam). CK8 staining was visualized using a DAB substrate kit for peroxidase from Vector Laboratories and counterstained and mounted as described above. Digital photographs at 4× and 10× were taken, and Image J software was used to quantify the ratios of the invaded vessel to the amount vessels in wildtype and *Cmklr1* deficient decidual zone.

### Serum IL-15 Measurement

The blood from wildtype and *Cmklr*1^−/−^ mice in GD 12 were collected. Serum was collected and frozen at −20°C in aliquots. According to the manufacturer’s protocol, serum IL-15 levels were measured using commercially available and sandwich enzyme-linked immunosorbent assay (ELISA) kits (R&D Systems, CA, United States). The samples were analyzed in triplicate.

### Statistical Analysis

All values were expressed as mean ± SD. One-way ANOVA on Rank test was used to test the statistical differences between groups. Parametric Student’s t-test or non-parametric Mann Whitney U test was used where appropriate as the post-test. GraphPad Prism 9.0 (GraphPad Software, La Jolla, CA, United States) was used for all statistical analyses. A *p*-value of less than 0.05 was considered significant.

## Results

### Chemerin-CMKLR1 Axis Regulates the Proliferation and Cytokine Secretion of Human dNK Cells

**FIGURE 1:**
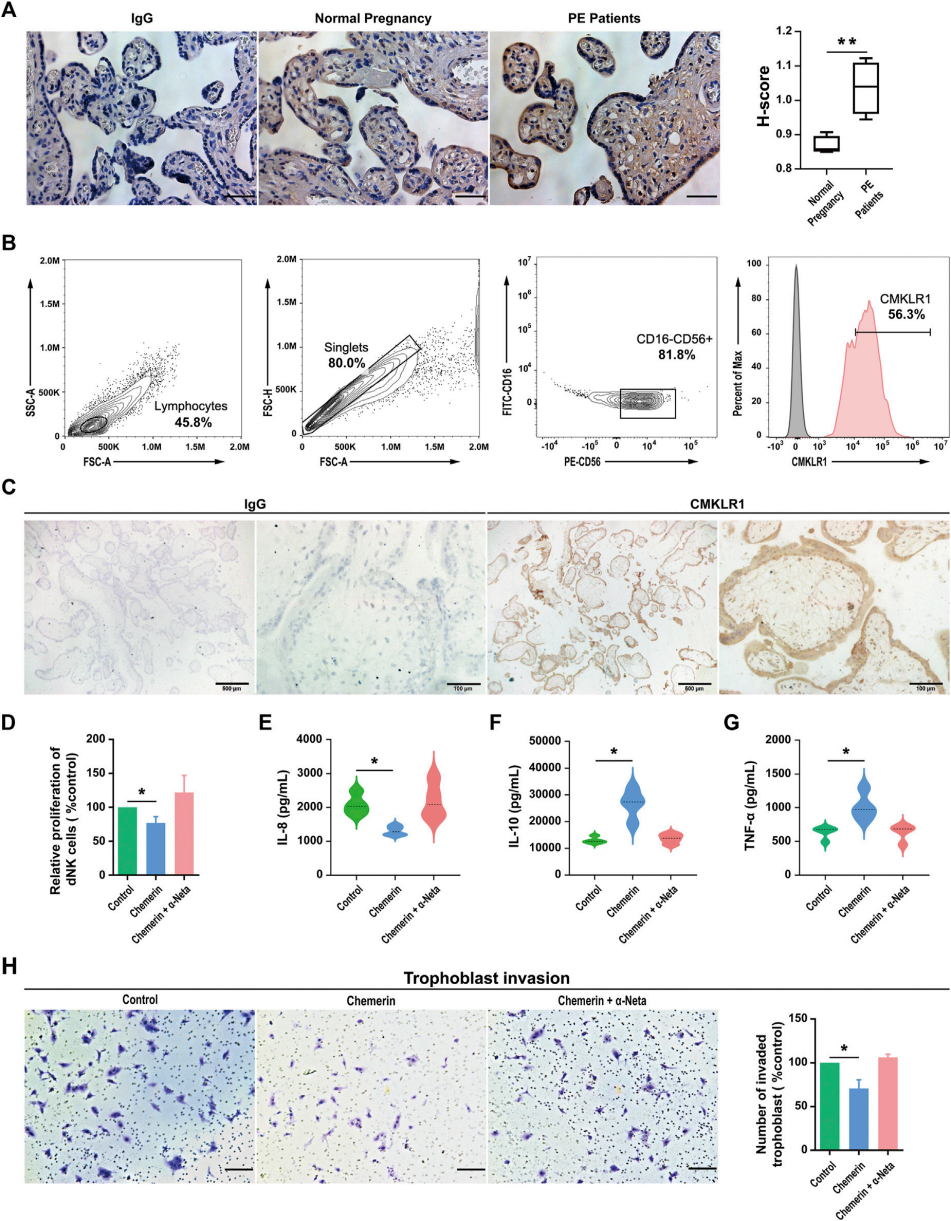
Chemerin-CMKLR1 axis regulates the proliferation and cytokine secretion of human dNK cells **(A)** Representative chemerin staining in placentas and the H-score of the area displaying chemerin positivity. Scale bar = 100 µm **(B)** The expression of CMKLR1 was detected in human dNK cells (CD56^+^CD16^−^) using flow cytometry **(C)** Location of CMKLR1 in human villi in early pregnancy based on immunohistochemistry using antibodies specific for CMKLR1. Brown color indicates specific CMKLR1 staining **(D)** The proliferation of primary dNK cells after being treated with 0.5 μM chemerin, 0.5 μM chemerin +1 µM α-Neta or vehicle for 24 h was assessed by proliferation kit. The concentration of IL-8 **(E)**, IL-10 **(F)** and TNF-α **(G)** in spent medium of primary dNK cells after being treated with 0.5 μM chemerin, 0.5 μM chemerin +1 µM α-Neta or vehicle were measured by ELISA. As isotype controls, sections were incubated with Isotype IgG control **(H)** The effect of spent medium from dNK cells treated with 0.5 μM chemerin, 0.5 μM chemerin +1 µM α-Neta on trophoblast invasion in a transwell assay. Invasive cells were stained with crystal violet. Scale bar = 200 µm. The invasive index was calculated as the percentage of invasion in treatment groups to the percentage of control group invasion. The results were represented as Mean ± SD, analyzed by One-way ANOVA; *p < 0.05 and **p < 0.01 vs. Control group; *N* = 5. dNK: decidual Natural Killer.

Chemerin immunoreactivities were positively stained in the trophoblast layer of human placental tissues. Compared with normal pregnancy, the expression of chemerin was reduced in the term pregnancy placental villi from women who presented with PE (Figure 1A). The biological effects of chemerin are mediated by CMKLR1. By using flow cytometry and immunohistochemistry, we demonstrated CMKLR1 expressions in CD16^−^CD56^+^ dNK (Figure 1B) cells and chorionic villi (Figure 1C) of first-trimester pregnancies respectively. To determine the role of CMKLR1 in regulating dNK cell proliferation, dNK cells were treated with chemerin with or without α-Neta, a specific antagonist of CMKLR1 for 24 h. Our results showed that while 0.5 µM chemerin suppressed the proliferation of the dNK cells, the inclusion of 1 µM α-Neta abolished the suppressive effect of chemerin (Figure 1D). The results indicated that the interaction of chemerin-CMKLR1 inhibited dNK cell proliferation.

The dNK cells are in close proximity to EVTs and regulate EVT functions by secretory factors. For example, dNK cell-derived IL-6, IL-8, hepatocyte growth factor, granulocyte-macrophage colony-stimulating factor (GM-CSF), and interferon-inducible protein-10 have been demonstrated to regulate human EVT motility and/or invasion (Hanna et al., 2006; Wallace et al., 2012). Therefore, we investigated the possible roles of chemerin on IL-8, IL-10 and TNF-α secretions by dNK cells. Our data revealed that IL-8 (Figure 1E) production was downregulated, while IL-10 (Figure 1F) and TNF-α (Figure 1G) were upregulated in dNK cells after chemerin treatment. The inclusion of 1 µM α-Neta abolished the effect of chemerin, indicating the involvement of CMKLR1.

To further confirm the implication of chemerin-CMKLR1 interaction in the regulatory activities of dNK cells on EVT functions, the spent medium of dNK cells after chemerin treatment was collected and applied to the EVTs. The dNK cells without chemerin treatment or with chemerin treatment in the presence of α-Neta were served as control. Our results showed that the spent medium of chemerin-treated dNK cells significantly decreased EVT invasion when compared to the controls, and such effect was abolished by α-Neta (Figure 1H).

### Chemerin-CMKLR1 Interaction Promotes Human Trophoblast Syncytialization

**FIGURE 2:**
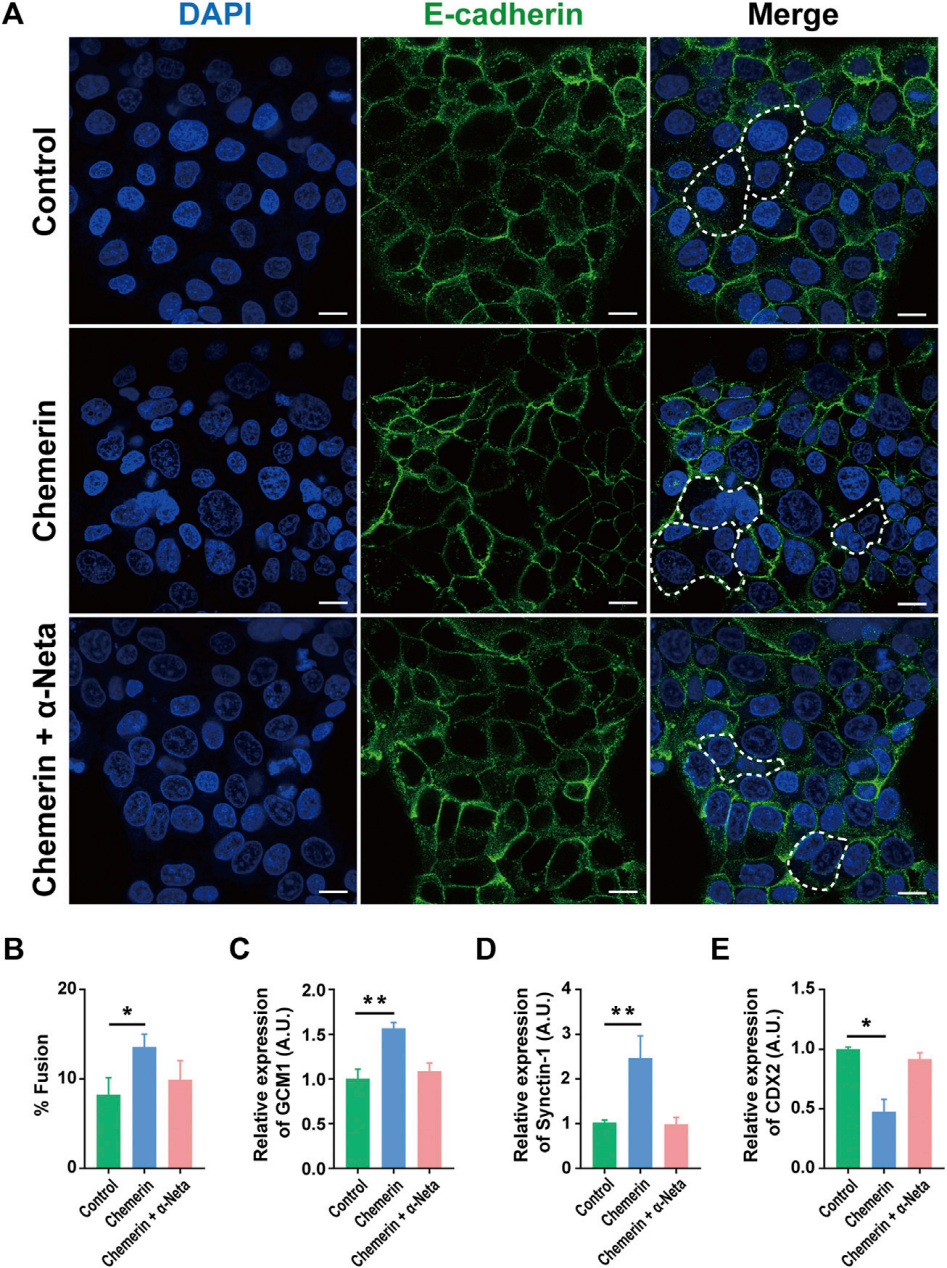
Chemerin-CMKLR1 interaction promotes human trophoblast syncytialization **(A)** Immunofluorescence staining of BeWo cells was also used to quantify BeWo cell fusion under the different stimulatory conditions. There was a low level of spontaneous fusion of BeWo cells, but most cells were in a mononucleated state. Chemerin stimulated the fusion of BeWo cells while the presence of chemerin in combination with α-NETA showed that chemerin-induced cell fusion was inhibited by α-NETA **(B)** Fusion index of BeWo cells treated with the vehicle, 0.5 µM chemerin and 0.5 µM chemerin +1 µM α-Neta for 48 h **(C)** The relative expression of trophoblast differentiation maker GCM1 **(D)** syncytiotrophoblast markers Synctin-1 were quantified by quantitative real-time PCR **(E)** The relative expression of trophectoderm marker CDX2 was analyzed by real-time PCR. The results were represented as Mean ± SD, analyzed by One-way ANOVA; *p < 0.05 and ***p < 0.001 vs. Control group; *N* = 5. GCM1: Human Glial Cells Missing-1; Synctin-1; CDX2: caudal-type homeobox gene 2. Scale bar = 100 µm.

Fusion of the cytotrophoblasts forms the multinucleated syncytiotrophoblast responsible for hormone production and maintenance of homeostasis of pregnancy. By using BeWo cells, an established model of forskolin induced syncytialization of cytotrophoblasts (Supplementary Figure S1), we demonstrated that chemerin-CMKLR1 interaction reduced E-cadherin expression, and thereby promoted syncytialization (Figures 2A,B). The inclusion of 1 µM α-Neta abolished the stimulatory effect of chemerin on syncytialization, indicating the involvement of CMKLR1 in cytotrophoblast syncytialization. The observations were supported by the upregulation of GCM1 (a trophoblastic differentiation marker) and Synctin-1 (a syncytiotrophoblast marker), and downregulation of CDX2 (a trophectoderm marker) in BeWo cells after chemerin treatment.

### *Cmklr1* Knockout Affects the Pregnancy Outcome *in Vivo*

**FIGURE 3:**
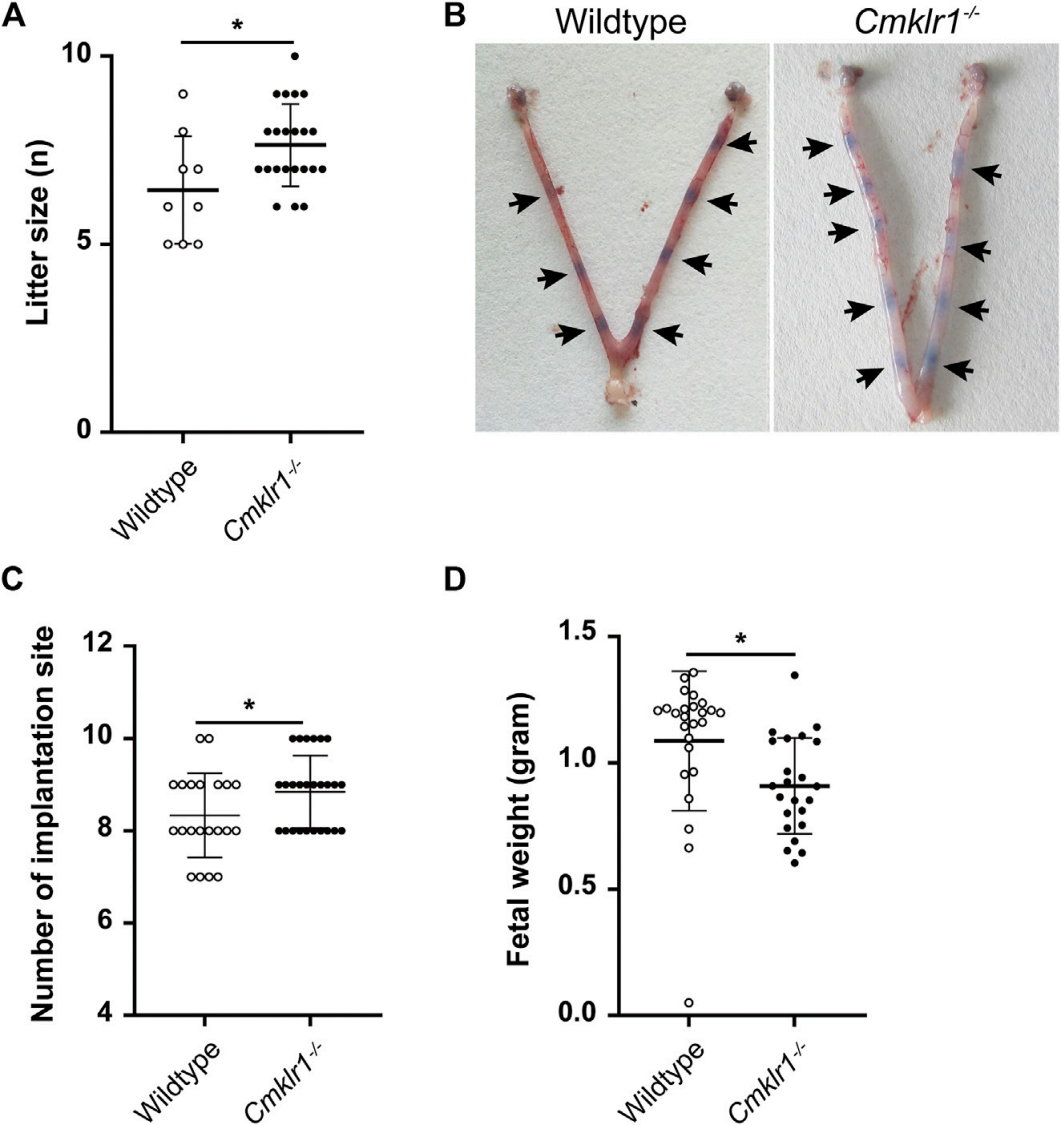
*Cmklr1* knockout affects the pregnancy outcome *in vivo* **(A)** Quantification of average litter size (*N* = 20 pregnant mice/genotype) **(B)** Representative embryos within uteri in wildtype and *Cmklr1* ^*−/−*^ mice at GD5. Arrowheads indicate viable implantation sites visualized as blue bands by uptake of Chicago Blue dye **(C)** Quantification of average implantation sites at GD5 (*N* = 20 pregnant mice/genotype) **(D)** The fetus weight at birth from wildtype mice and *Cmklr1* ^*−/−*^ mice. The results were represented as mean ± SD, *n* = 5. *p < 0.05. SD: standard deviation.

To assess the role of *Cmklr1* in placental development *in vivo*, a *Cmklr1*-deficient mouse model was generated (Supplementary Figure S2A). Successful ablation of *Cmklr1* was confirmed by PCR analysis (Supplementary Figure S2B). *Cmklr1* knockout had no effect on chemerin expression (Supplementary Figure S2C). The *Cmklr1* deficient mice exhibited larger litter size (Figure 3A) and more implantation sites (Figures 3B,C) but reduced fetal weight at birth (Figure 3D) when compared to the wildtype mice.

### *Cmklr1* Knockout Affects Placental Development and Spiral Arterial Remodeling *in Vivo*

**FIGURE 4:**
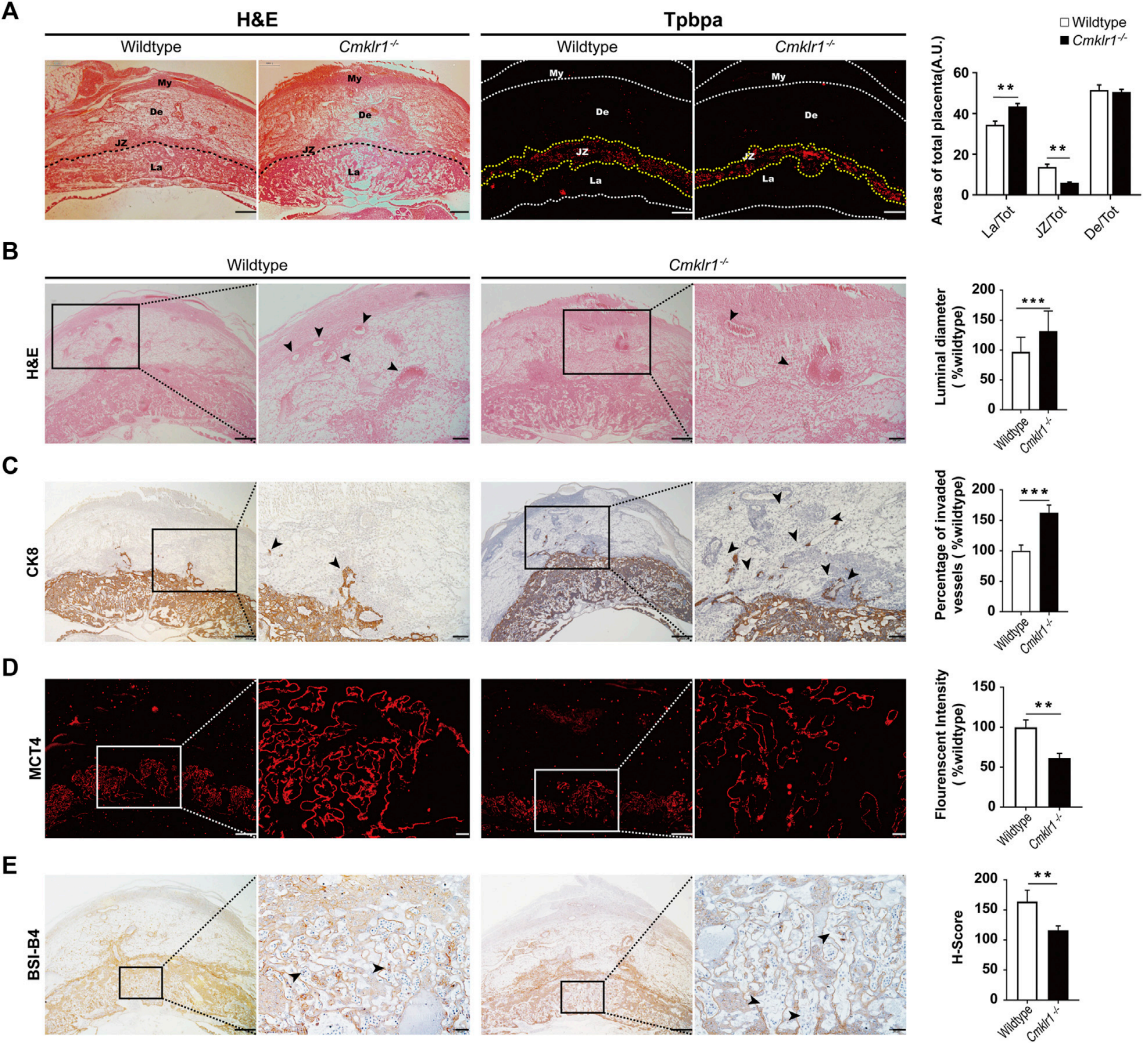
*Cmklr1* knockout affects the placental development and spiral arterial remodeling *in vivo* **(A)** Representative H&E-stained transverse sections of placentas. The immunofluorescent staining of TPBPA for outlining the junctional zone. The ratio of the junctional zone, labyrinth layer, and decidual zone to total placental areas **(B)** H&E staining of placental sections from wildtype mice and *Cmklr1* ^*−/−*^ mice at GD12 in different magnifications. Quantification of the spiral arterial luminal diameter. The middle placenta section from each litter (four litters from four mothers per group) was used for analysis. The spiral arteries are indicated by arrowheads **(C)** The invasion ability of the trophoblast was examined. Transverse sections of wildtype and *Cmklr1* ^*−/−*^ placentas at GD12 were immunohistochemically stained with antibodies against trophoblast marker CK8 in different magnifications. Invading trophoblasts are indicated by arrowheads. The trophoblast invaded vessels percentage was analyzed in 3 mid-sagittal implantation site sections per mouse (*n* = 4/group) and averaged using ImageJ software. Quantification of the ratios of the invaded vessel to the amount vessels in wildtype and *Cmklr1* ^*−/−*^ decidua zone **(D)** Immunofluorescent staining with MCT4 showing the syncytiotrophoblast II in the transverse sections of placentas in wildtype and *Cmklr1* ^*−/−*^ mice at GD 12 in different magnifications. Quantification of the MCT4 expression in the labyrinth layers in wildtype and *Cmklr1* ^*−/−*^ placentas **(E)** Immunohistochemistry with isolectin BSI-B4 staining shows the fetal blood vessels in the transverse placenta’s sections in wildtype and *Cmklr1* ^*−/−*^ mice at GD 12 in different magnifications. H-score of BSI-B4 positive staining in the labyrinth trophoblasts around fetal blood vessels in wildtype and *Cmklr1* ^*−/−*^ placentas. The maternal lacunae are indicated by arrowheads. The middle placenta section from each litter (four litters from four mothers per group) was used for analysis. The results were represented as the mean ± SD. *p < 0.05, **p < 0.01. Scale bar = 200 μm. BS1: isolectin BSI-B4 (GSL I-B4); MCT4: Monocarboxylate transporter 4; SD: standard deviation.

Cellular morphology of the wildtype and the *Cmklr1*-deficient placentas were compared (Figure 4A) which showed a significant reduction of the junctional zone thickness as indicated by TPBPA staining, and thus a proportionally increased labyrinth zone in the *Cmklr1*-knockout placentas. Significant enlargement of the luminal diameter of the spiral artery was also observed in the *Cmklr1* ^*−/−*^ mice (Figure 4B) which was accompanied by accumulation of the CK8 positive trophoblast (Figure 4C). In contrast, there was no difference in size of the decidua basalis between the *Cmklr1* ^*−/−*^ and the wildtype pregnant mice (Figure 4A).

To determine syncytialization in the *Cmklr1*-knockout placenta, we stained for MCT4, a monocarboxylate transporter specifically expressed in the syncytiotrophoblast II layer. The thickness of the syncytiotrophoblast II layer and the MCT4 staining were significantly reduced in the *Cmklr1*-deficient labyrinth. As the syncytiotrophoblast II layer enclosed fetal blood vessels in the labyrinth, the reduced MCT4 staining suggested that the fetal blood vessels were not properly developed in the region (Figure 4D), consistent with the diminished BSI-B4 staining that outlines the matrix surrounding the fetal vessels in the *Cmklr1*-deficient placenta when compared to the wildtype (Figure 4E).

### *Cmklr1* Knockout Is Associated With Increased dNK Cells

**FIGURE 5:**
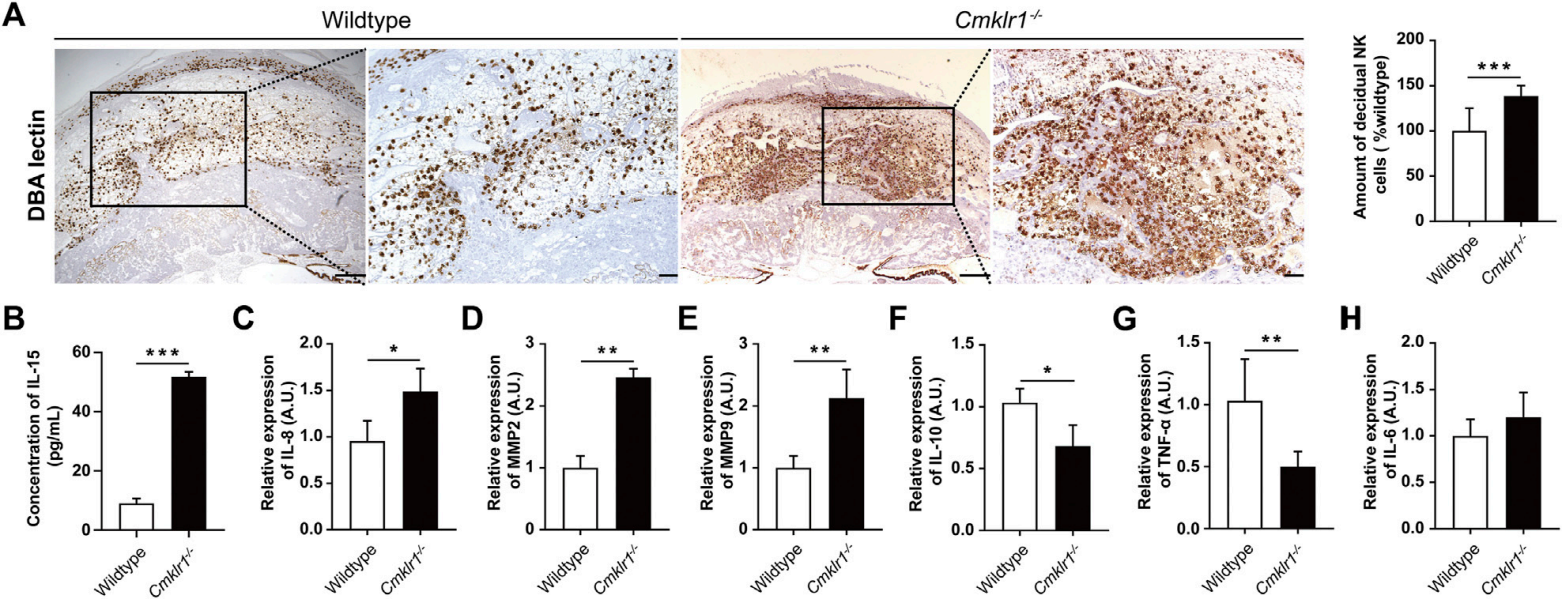
*Cmklr1* knockout is associated with increased dNK cells. **(A)** The amount and distribution of dNK cells of wildtype and *Cmklr1* ^*−/−*^ were investigated by immunohistochemically staining with antibodies against DBA Lectin in different magnifications. Scale bar = 200 μm. Quantification of the number of dNK cells. **(B)** The concentration of IL-15 in serum of wildtype and *Cmklr1* ^*−/−*^ at GD 12 was measured by ELISA. Total RNA samples from wildtype and *Cmklr1* ^*−/−*^ placentas at GD12 were subjected to qRT-PCR. The middle placenta section from each litter (four litters from four mothers per group) was used for analysis. The expression of **(C)** IL-8, **(D)** MMP2, **(E)** MMP9, **(F)** IL-10, **(G)** TNF-α and **(H)** IL-6 were examined. The results were represented as mean ± SD, *n* = 4. *p < 0.05 and **p < 0.01. IL: Interleukin; TNF-α: Tumor necrosis factor-alpha; MMP: Matrix Metallopeptidase; TIMP: Tissue inhibitor of metalloproteinases. qRT-PCR: quantitative reverse-transcription polymerase chain reaction; SD: standard deviation. TPBPA: trophoblast-specific protein alpha.

Successful placental development and spiral arterial remodeling require dNK cells. The *Cmklr1* ^*−/−*^ mice had a significant increase in the number of DBA-lectin positive dNK cells (Figure 5A) in the decidual zone when compared to the wildtype. IL-15 is involved in activating the proliferation of the CD56^Bright^ NK cells (Felices et al., 2018). Therefore, we analyzed the serum IL-15 level of the *Cmklr1* ^−/−^ pregnant mice at GD 12. We detected a 5-fold increment in serum IL-15 level in the *Cmklr1* ^−/−^ mice when compared to the wildtype (Figure 5B). We also investigated the downstream factors responsible for the altered placental development in the *Cmklr1-*knockout placenta. These factors, including IL-6 (Champion et al., 2012), IL-8 (Jovanović et al., 2010), IL-10 (Pang et al., 2008), MMP2, MMP9 (Staun-Ram et al., 2004), and TNF-α have been associated with the regulatory activities of dNK cells on trophoblast functions. Our data revealed that IL-8 (Figure 5C), MMP2 (Figure 5D), and MMP9 (Figure 5E) were upregulated while IL-10 (Figure 5F) and TNF-α (Figure 5G) were downregulated. The expression of IL-6 was not significantly affected (Figure 5H).

## Discussion

**FIGURE 6:**
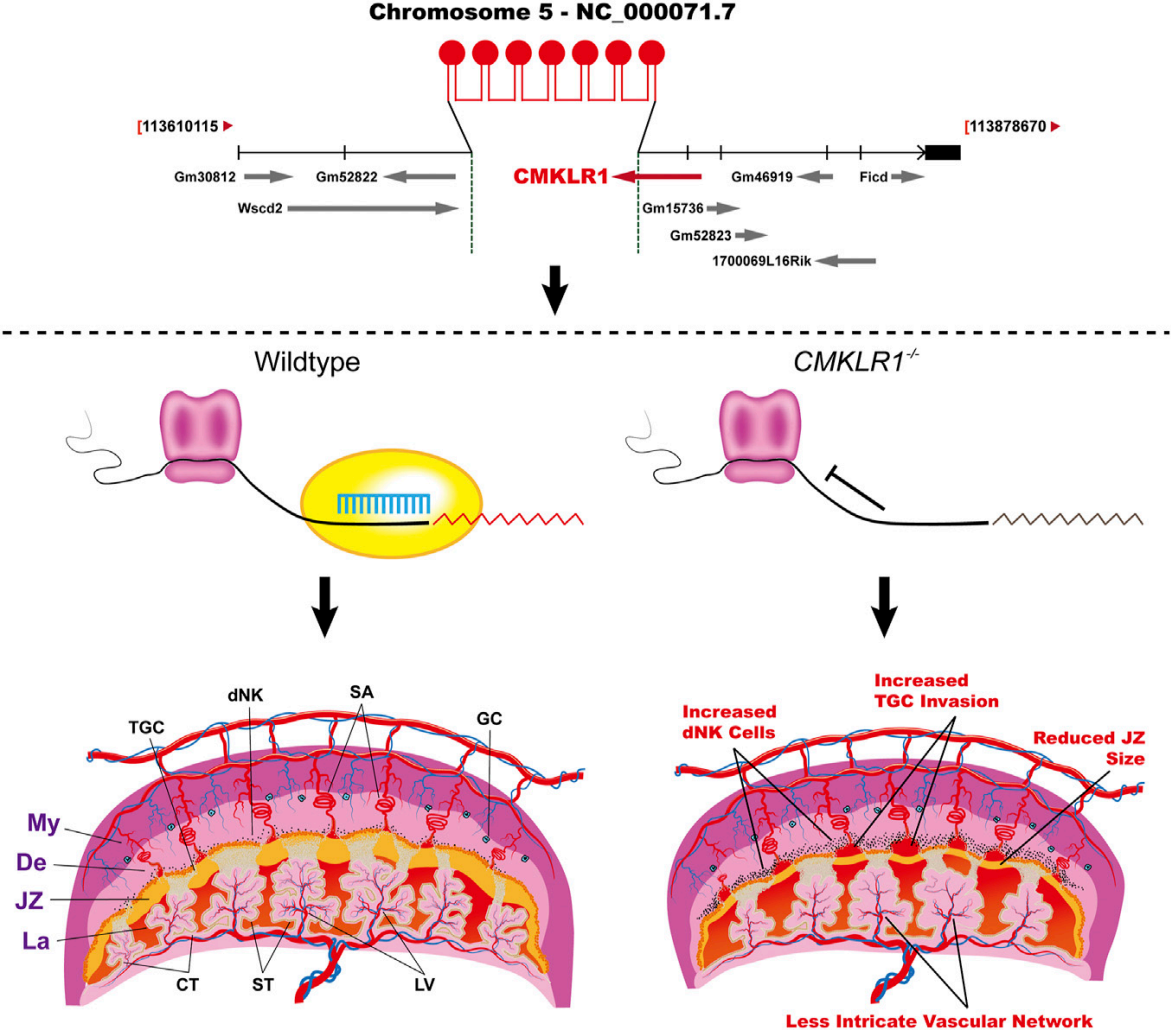
The working model shows the regulation of CMKLR1 signaling during placental development. Upper: The strategy of *Cmklr1* gene knockout in mouse. Lower: *Cmklr1* ^*−/−*^ led to increased trophoblast invasion by increased proliferation of dNK cells and reduced JZ by reduced syncytiotrophoblast II differentiation. My: myometrium; De: decidua; JZ: junctional zone; La: Labyrinth; TGC: trophoblast giant cells; dNK: decidual Natural Killer; SA: spiral artery; GC: giant cells; CT: chronic trophoblast; ST: syncytiotrophoblast; LV: labyrinth vasculature.

The increased placental chemerin level was related to human PE and overexpression of chemerin in trophoblast contributed to the development of PE-like symptoms via its receptor CMKLR1 (Tan et al., 2022). Here, to elucidate the role of chemerin-CMKLR1 in pregnancy, we generated mice knockout for *Cmklr1*. We found that the weight of *Cmklr1* ^*−/−*^ fetus at birth was reduced by around 15%. It could be speculated that the reduced fetus growth was related to defective placental development. During labyrinth formation, the syncytiotrophoblast layers facilitate fetomaternal exchanges by intercrossing between the fetal vessel and maternal blood lacunae (Dupressoir et al., 2011). The extent of the trophoblast branch contributes to blood vessels in the mature labyrinth development (Watson and Cross, 2005). Here we demonstrated that the *Cmklr1* ^−/−^ mice showed 1) an increased total labyrinth area with enlarged maternal blood lacunae; 2) degenerative changes in interhemal trophoblasts; 3) defects in syncytial formation of the syncytiotrophoblast II, facing the embryonic vessels, which was confirmed by the ability of α-Neta in abolishment of observed effect on syncytialization. The defective syncytiotrophoblast II development may attenuate the interactions and functions of neighboring trophoblast cells leading to the observed reduced interhemal trophoblasts and disorganization of the maternal-fetal transferring network in the labyrinth, and ﬁnally to reduced fetal growth. As summarized above, we proposed the working model for CMKLR1 in placental development (Figure 6).

Patients suffering from hypertension and PE showed significantly higher serum chemerin concentrations (Cetin et al., 2017; Xie and Liu, 2022). The increased placental chemerin was also found in the villi of preeclamptic women (Xu et al., 2014; Tan et al., 2022). Our findings demonstrated that *Cmklr1* knockout mice had more invaded trophoblasts and larger arteries in the decidua and myometrium than those in wildtype mice. Consistently, overexpression of trophoblastic chemerin diminishes trophoblast invasion (Tan et al., 2022). These observations indicate that CMKLR1 mediates the role of chemerin in pregnancy.

Trophoblast invasion and dNK cell functions are indispensable for spiral artery remodeling (James et al., 2010). During placental development, dNK cells (CD56^bright^CD16^−^) are the predominant lymphocyte population (around 70–80%) at the implantation sites (Liu et al., 2017), where trophoblast invasion and vascular remodeling happen (Hanna et al., 2006). Our data showed that CMKLR1 was expressed in the CD16^−^CD56^bright^ NK cells, in line with a previous report (Carlino et al., 2012). The interaction of chemerin-CMKLR1 inhibited dNK cell proliferation and decreased EVTs invasion. These are in line with our *in-vivo* study showing increased trophoblast invasion deeply into the spiral artery in the *Cmklr1*-knockout mice placenta. The regulation of trophoblast invasion at the maternal-fetal interface is controlled by various factors, including cytokines, chemokines, growth factors, sex hormones, critical protease, and adhesion molecules (Harris et al., 2009) through autocrine or paracrine action (Bischoff et al., 2000). These factors are secreted from the trophoblast, decidual endothelial cells and dNK cells, suggesting a complex network to regulate trophoblast invasiveness (Knöfler, 2010). Based on the increased dNK cell number in the mouse placenta and the increased proliferation of human dNK cells treated with chemerin, the deep trophoblast invasion of the *Cmklr1* ^*−/−*^ mice placentas may be regulated by secretions of the dNK cells.

The dNK cells are well known to express killer cell immunoglobulin-like receptors and produce angiogenic cytokines and proteins such as TNF-α, interferon-γ, and vascular endothelial growth factor (VEGF) that regulate trophoblastic invasion, angiogenesis, and vascular remodeling (Ashkar et al., 2000; Fukui et al., 2012). The dNK cells also produce MMP2 and MMP9, which together with VEGF, placental growth factor (PGF), and angiopoietin are essential for maintenance of vessel stability (Smith et al., 2009). Consistently, both our *in vitro* and *in vivo* studies confirmed that chemerin-CMKLR1 axis is involved in regulating the productions of trophoblast regulatory factors from dNK cells, including TNF-α, IL-10 and IL8.

We observed upregulation of IL-15 in the *Cmklr1* ^−/−^ mice serum. IL-15 plays a role in development, survival, and activation of the NK cells, homeostasis of natural killer T (NKT) cells and intraepithelial lymphocytes, and maintenance of the naïve and memory CD8^+^ T cells (Koka et al., 2003). IL-15 and IL-15Rα-deficient mice lack NK cells and have severely reduced numbers of NKT cells, memory CD8^+^ T cells, and specific subsets of intestinal intraepithelial lymphocytes (Koka et al., 2003; Terabe et al., 2008). The IL15^−/−^ mice exhibited impaired remodeling of the spiral arteries, including decreased lumen diameters and thicker vessel walls (Ashkar et al., 2003). IL-15 might also participate in cytotrophoblast invasion, possibly related to the increased expression of MMP-1(Sharma et al., 2016). IL-15 is secreted by a large variety of tissues and cell types. Its expression in the endometrium is stronger during the secretory phase and first trimester pregnancy when compared to the proliferative phase, consistent with involvement of IL-15 in early placentation (Kitaya et al., 2000). Since IL-15 is also produced by placenta (Li et al., 2021), the increased serum IL-15 level in *Cmklr1* knockout mice may be contributed by both decidua and placental tissues. This IL-15 upregulation may activate the dNK cells and thereby modulate the trophoblast functions. These observations provide the cellular mechanism on how the dNK cell proliferation is related to the increased trophoblast invasion in the *Cmklr1* ^−/−^ mice.

In summary, CMKLR1 is required for placental development. The deletion of CMKLR1 decreased fetal vessel density, syncytiotrophoblast II development and enlarged maternal lacunae in the labyrinth while increased trophoblast invasion and enlarged spiral artery lumen in the decidua. The possible reasons for the observed beneficial effect of *Cmklr1* knockout on spiral artery remolding are increase in dNK cell proliferation and IL-15 production, upregulation of MMP2, MMP9, and reduction of TNF-α and IL-10, subsequently enhancement of trophoblast invasion. A major limitation of this study is the suppression of CMKLR1 is not placenta-specific, and the loss of CMKLR1 in other organs might have a confounding effect on the development of the placenta. Another limitation is that there is potential interference caused by the presence of endogenous chemerin from dNK cells in the *in vitro* experiments which might affect the result interpretation. Also, the specific CMKLR1 signaling cascade responsible for causing the phenotype is still unknown. Further investigation is required to define the exact molecular mechanisms how chemerin elevation can contribute to PE. It may associate with the dysregulation of transcription factor HOXA9 which has been reported to activate chemerin transcription, promote pyroptosis and inflammation of trophoblasts, and contributed to PE (Quan et al., 2021). The results of this study suggest that CMKLR1 expression may be used for the early prediction of placenta-associated complications with altered NK cell biology. Suppression of CMKLR1 via a specific antagonist could be a possible treatment for PE.

## Data Availability

The original contributions presented in the study are included in the article/Supplementary Material, further inquiries can be directed to the corresponding authors.
