# Supplementary material for: The Regulatory Roles of Chemerin-Chemokine-Like Receptor 1 Axis in Placental Development and Vascular Remodeling During Early Pregnancy
Source: Front Cell Dev Biol. 2022 May 17;10:883636. doi: 10.3389/fcell.2022.883636 (PMC9152263; doi:10.3389/fcell.2022.883636)
Supplement: Supplementary file 1 [file DataSheet1.PDF]

Supplementary Material

1.1 Supplementary Figures

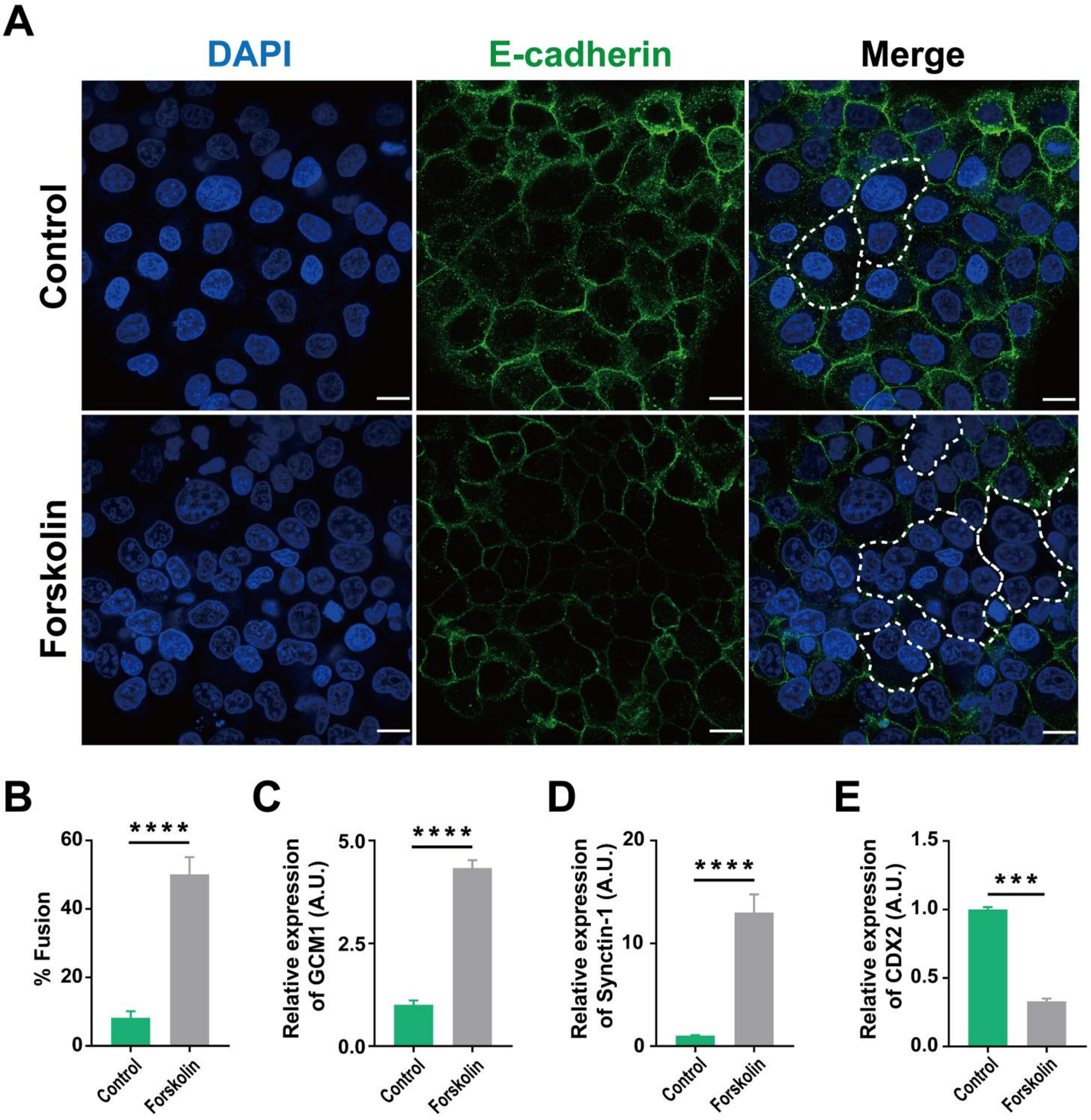

Supplementary Figure 1. The effect of forskolin on the syncytiotrophoblast fusion and differentiation. (A) There was a low level of spontaneous fusion of BeWo cells, but most cells were in a mononucleated state. When BeWo cells were treated with forskolin, cell borders and the E-cadherin staining vanished in the infusing cells (B) Fusion index of BeWo cells treated

with the vehicle and 20 µM Forskolin (as positive control) for 48 hours. (C) The relative expression of trophoblast differentiation maker GCM1, (D) syncytiotrophoblast markers Synctin-1 were quantified by quantitative real-time PCR. (E) The relative expression of trophectoderm marker CDX2 was analyzed by real-time PCR. The results were represented as Mean ± SD, analyzed by One-way ANOVA; ***$p < 0.001$ vs. Control group; N = 5. GCM1: Human Glial Cells Missing-1; Synctin-1; CDX2: caudal-type homeobox gene 2.

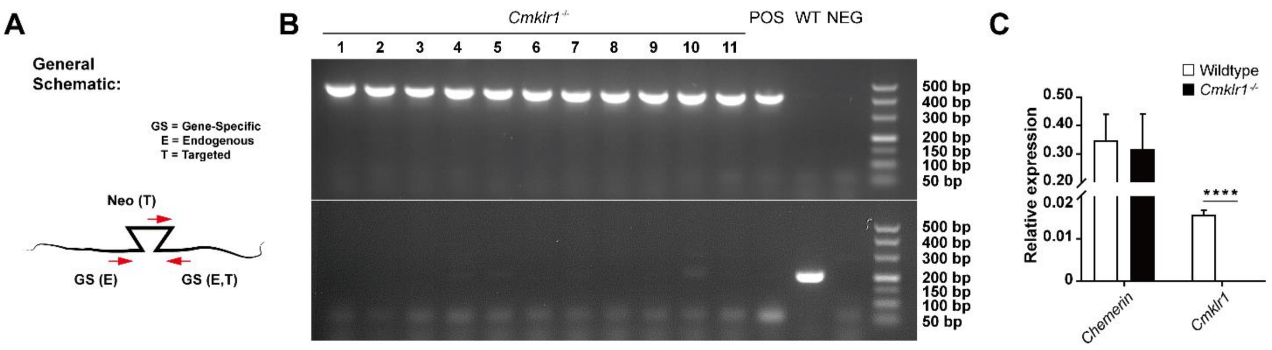

Supplementary Figure 2. *Cmklr1* knockout placentas have no effect on chemerin expression. (A) Schematic of *Cmklr1* knockout strategy. (B) Validation of *Cmklr1* knockout by PCR analysis. A 424 bp PCR product verifies insertion of Neo cassette by primers GS(E) forward and GS (E, T) reverse. Wildtype mice will have 205 bp PCR products as amplified by primer GS(E) forward and GS (E, T) reverse. (C) Expression of chemerin and *Cmklr1* were detected by Q-PCR analysis in mouse placenta p on Gestation Day GD 12 in wildtype and *Cmklr1*-null mice.
